# A Novel Domain Regulating Degradation of the Glomerular Slit Diaphragm Protein Podocin in Cell Culture Systems

**DOI:** 10.1371/journal.pone.0057078

**Published:** 2013-02-20

**Authors:** Markus Gödel, Benjamin N. Ostendorf, Jessica Baumer, Katrin Weber, Tobias B. Huber

**Affiliations:** 1 Renal Division, University Hospital Freiburg, Freiburg, Germany; 2 BIOSS Centre for Biological Signalling Studies, Albert-Ludwigs-University Freiburg, Freiburg, Germany; University of Houston, United States of America

## Abstract

Mutations in the gene *NPHS2* are the most common cause of hereditary steroid-resistant nephrotic syndrome. Its gene product, the stomatin family member protein podocin represents a core component of the slit diaphragm, a unique structure that bridges the space between adjacent podocyte foot processes in the kidney glomerulus. Dislocation and misexpression of slit diaphragm components have been described in the pathogenesis of acquired and hereditary nephrotic syndrome. However, little is known about mechanisms regulating cellular trafficking and turnover of podocin. Here, we discover a three amino acids-comprising motif regulating intracellular localization of podocin in cell culture systems. Mutations of this motif led to markedly reduced degradation of podocin. These findings give novel insight into the molecular biology of the slit diaphragm protein podocin, enabling future research to establish the biological relevance of podocin turnover and localization.

## Introduction

Podocytes are specialized epithelial cells constituting an essential part of the glomerular filtration barrier. They form a delicate network of cell extensions, so called primary and secondary processes that enwrap the glomerular capillaries. Interdigitating secondary processes are connected by a specialized cell junction, the slit diaphragm. Orderly composition of the slit diaphragm is essential for various cellular functions of the podocyte such as cell survival, polarity and cytoskeletal organization [1], [2]. Over the last decade, much progress has been made in identifying the molecular make-up of the slit diaphragm [3]–[5].

*NPHS2* is the most frequently affected gene in steroid-resistant nephrotic syndrome. Mutations in *NPHS2* are responsible for about 50% of familial (autosomal recessive) and up to 20% of sporadic cases [4], [6], [7]. So far, expression of its gene product, the PHB-domain containing protein podocin, has only been shown in the glomerular podocyte and testis Sertoli cells [8]. In the podocyte, podocin localizes to the slit diaphragm, where it is assumed to act as an intracellular scaffold protein, assembling slit diaphragm components in lipid raft associated microdomains [9], [10]. Podocin is a membrane-attached protein. It is predicted to form a hairpin like structure, with both N- and C-terminus residing in the cytoplasm. Several disease causing *NPHS2* mutations were shown to interfere with podocin intracellular trafficking [11].

Various forms of injury to the glomerular filter trigger a common pathophysiological pathway inducing podocyte foot process effacement. Subcellularly, effacement is accompanied by the dislocation and degradation of slit diaphragm associated proteins such as nephrin and podocin [12]–[14]. It is therefore assumed that the spatiotemporal regulation of slit diaphragm components plays an essential role in the homeostasis of glomerular function [15]. The comprehensive knowledge of molecular events affecting slit diaphragm stability and degradation will be helpful in identifying novel therapies to maintain function and size selectivity of the glomerular filter in nephrotic disease. Recently, mechanisms such as ubiquitination and phosphorylation have been shown to participate in regulating nephrin endocytosis and degradation [16]–[19]. However, despite its significance at the slit diaphragm the mechanisms regulating the turnover of podocin remain unknown. It was therefore the aim of this work to investigate into these mechanisms in order to provide the basis for future research projects defining the biological relevance of podocin turnover and localization for podocyte physiology. Using a cell culture-based approach we were able to map a three amino acids comprising domain influencing subcellular localization and subsequent degradation of podocin.

## Materials and Methods

### Reagents and Plasmids

Murine podocin, human transferrin-receptor and pLXSN plasmids have been described previously [10], [20], [21]. All truncated or mutated variants of podocin were generated using standard cloning techniques. Solely N-terminally tagged fusion constructs of podocin (Flag, V5) were used for this study. Fusion proteins of podocin with a CD7-CD16 header were generated using a vector kindly provided by G. Walz [22]. A cDNA construct encoding eGFP-CD63 was kindly provided by D. Cutler. All newly synthesized constructs were verified by automated sequencing. For immunofluorescence, primary antibodies were obtained from Santa Cruz (anti-CD16 mouse mAb, sc-51525), Sigma (anti-Flag rabbit pAb, F7425), Serotec (anti-V5 mouse mAb, MCA-1360), Chemicon Millipore (anti-V5 rabbit pAb, AB3792), Cell Signaling (anti-EEA1 rabbit pAb, 2411; anti-calnexin rabbit pAb, 2433) and Molecular Probes (anti-golgin97 mouse mAb, A-21270). Nuclear staining reagents and fluorophore-conjugated secondary antibodies were obtained from Invitrogen (Hoechst 33342, H3570; Alexa Fluor 488 donkey anti-rabbit, A21206; Alexa Fluor 488 donkey anti-mouse, A21202; Alexa Fluor 555 donkey anti-mouse, A31570; Alexa Fluor 555 donkey anti-rabbit, A31572). Lysotracker Red DND-99 was obtained from Invitrogen (L-7528). For western blot, antibodies were obtained from Sigma (anti-Flag mouse mAb, F3165; anti-actin mouse mAb, A1978) and HRP-conjugated secondary antibodies were obtained from Dako.

### Cell culture

HEK 293T and HeLa cells were grown in Dulbecco's modified Eagle's medium (DMEM) supplemented with 10% FBS. A human podocyte cell line was provided by M. Saleem (Children's Renal Unit, Bristol Royal Hospital for Children, University of Bristol, UK) and was cultured as described previously [23].

### Generation of Transgenic Podocyte Cell Lines

Transgenic podocyte cell lines were generated using the pLXSN-vector expression system as previously described [21]. Briefly, retrovirus was produced by transfection of HEK 293T cells with 2.5 µg of pMD-G, 7.5 µg of pMD-gp and 10 µg of the retroviral transfer vector pLXSN. The supernatant was harvested, centrifuged, filtered and cultured podocytes were transduced three times.

### Immunofluorescence and Protein Internalisation

HeLa cells were transfected using Lipofectamine 2000 by Invitrogen and stained as described previously [10]. Briefly, HeLa cells or podocytes were fixed in 4% PFA for 3 minutes or in methanol at −20°C for 10 minutes when using anti-Flag antibody. They were then washed with PBS, permeabilised with 0,01% Triton-X in PBS and blocked using 5% BSA in PBS. Incubation periods with primary and secondary antibodies were followed by multiple washing steps after which the cells were mounted in Prolong Gold Antifade (Invitrogen) and subjected to immunofluorescence microscopy using an Apotome microscope (Zeiss).

To label acidic organelles cells were incubated with 50 nM Lysotracker Red DND-99 for 35 minutes at 37°C, 5% CO_2_, washed twice with room temperature PBS, followed by fixation in 4% PFA for 10 minutes. Afterwards immunofluorescence was performed as described above.

Transient overexpression in human podocyte cell culture was performed by using Lonza Nucleofector technology with the primary epithelial cell transfection kit. 2 µg of plasmid DNA were electroporated using Nucleofector I program T-20 according to the manufacturer's standard protocol.

To demonstrate protein internalization cells were serum starved for 1 hour and incubated with primary antibody at 4°C for 20 minutes (anti-CD16 mouse mAb, Santa Cruz (sc-51525) 1∶1000 in DMEM and 20 mM HEPES). Following three washings they were incubated at either 4°C for controls or at 37°C for 20 minutes. Antibody remaining extracellularly was stripped in a 2 minute incubation step (0.5% acetic acid, 0.5 M NaCl, pH 3) and cells were subsequently washed and fixed. Further treatment was performed as described above.

### Protein Stability Assay

Transgenic podocyte cells were treated with 20 µg/ml cycloheximide in DMEM for the times as indicated, lysed in buffer A (8 M urea, 100 mM NaH2PO4, 10 mM Tris, 1% Triton X-100, pH 8.0) and analyzed by western blot. Densitometry of western blots was performed using ImageJ software and podocin levels were normalized to actin levels.

### FACS

HEK 293T cells were seeded on poly-L-lysine coated slides (poly-L-lysine diluted 1∶1 with H_2_O; Sigma P4707, 0.01% solution) and transfected with CD16-tagged constructs of podocin also expressing GFP driven by an internal ribosomal entry site (IRES). At 24 hours after transfection cells were serum starved for one hour, cooled on ice for 15 minutes and incubated with anti-CD16 antibody (anti-CD16 mouse mAb, Santa Cruz (sc-51525) 1∶750 in DFH (10% FBS and 20 mM HEPES in DMEM)). Subsequent to extensive washing with DFH they were incubated with fluorophore-conjugated anti-mouse antibody for 20 minutes (anti-mouse Alexa 633, Invitrogen (A21050) 1∶500 in DFH), washed again, resuspended in 5 mM EDTA/PBS and separated with cell strainers (BD Biosciences). FACS analysis was performed using a FacsCalibur machine (BD Biosciences). Gates were set to include GFP-positive cells only.

### Lipid Raft Preparation

Lipid rafts were isolated by sucrose density centrifugation as described previously [10]. Briefly, HEK293T cells were transfected using the calcium phosphate method and homogenized by 20 strokes in a Dounce homogenizer in 850 µl of TNE buffer (130 mM NaCl, 5 mM EDTA, 20 mM Tris, pH 8.2, proteinase inhibitors) in the presence of 1% Triton X-100. The lysates were incubated on ice for 20 min and centrifuged for 10 min at 3000 g at 4°C. The supernatant was adjusted to 50% sucrose and pipetted at the bottom of an ultracentrifuge tube. Samples were then overlaid with a sucrose step gradient (2 ml of 30% sucrose and 1 ml of 5% sucrose in TNE). Gradients were centrifuged for 16 h at 200 000 g at 4°C in a swing-out rotor, and seven fractions (700 µl each) were collected starting from the top and analyzed by SDS–PAGE.

### Computational Protein Structure Prediction

Protein Structure Prediction was carried out using the I-TASSER algorithm [24].

### Statistics

Data are expressed as means ± standard errors of the mean (SEM) of n experiments. All experiments were performed at least 3 times. Data analysis was performed using GraphPad Prism 5 statistical software.

## Results

### Podocin Localizes to the Endosomal Compartment

**Figure 1:**
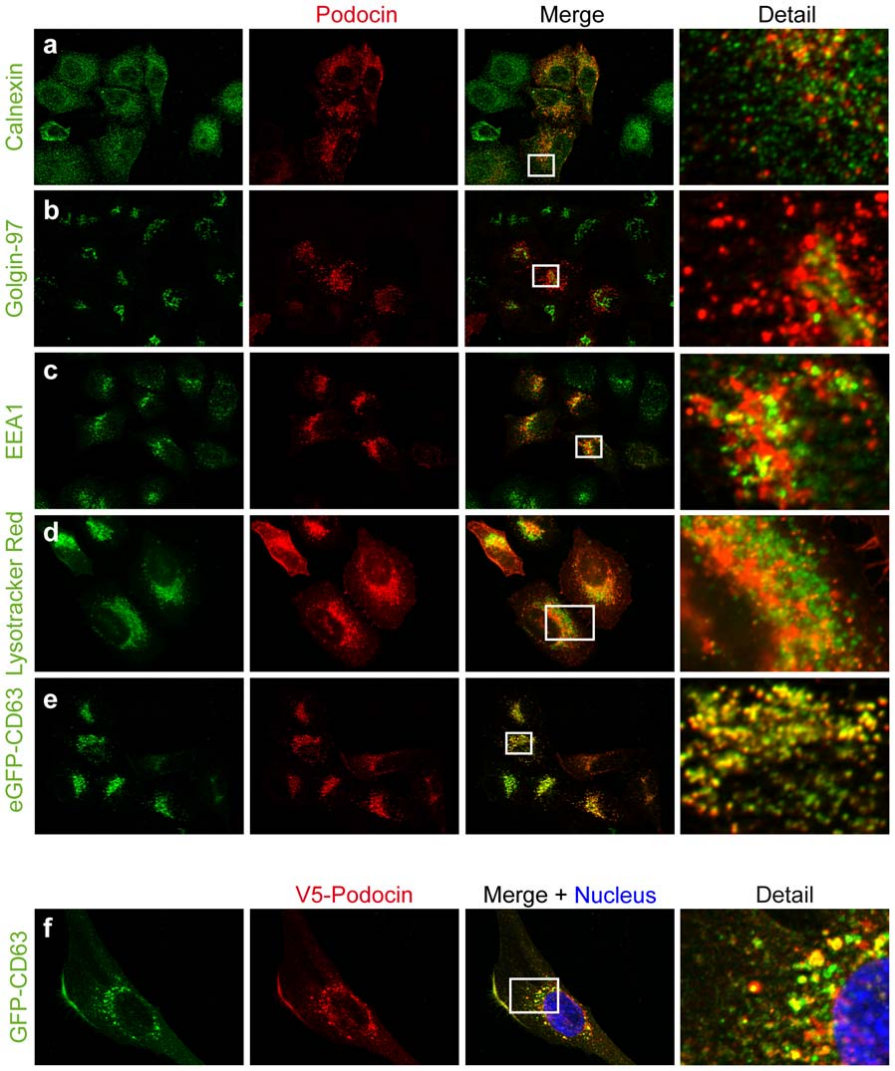
Podocin localizes to endosomal vesicles. Immunofluorescence for transiently expressed, V5- (a–c, e) or Flag-tagged (d) podocin using anti-V5 or anti-Flag antibody in HeLa cells. Costainings of endogenous calnexin (a), endogenous golgin-97 (b), endogenous EEA1 (c), Lysotracker Red (d) and eGfp-tagged and transiently expressed CD63 (e) as markers for the endoplasmatic reticulum, Golgi apparatus, early endosomes, acidic organelles and late endosomes respectively, displayed that podocin localizes to the endosomal compartment. Analogously to HeLa cells, transiently expressed V5-tagged podocin colocalizes with eGFP tagged CD63/LAMP3 in cultured human podocytes (f).

Previously, podocin has been shown to reside in intracellular, vesicle-like structures next to its canonic membranous localization [25]. Consistently, in addition to the localization to the plasma membrane, the immunofluorescence signal of V5-tagged podocin in transiently transfected HeLa-cells also revealed podocin localizing to intracellular vesicles. In order to characterize those structures we co-stained V5-tagged podocin with markers for different cellular compartments. Little colocalization could be observed with markers for the secretory pathway, calnexin and golgin-97 (Fig. 1a and b), which stain the endoplasmatic reticulum and Golgi apparatus respectively. Also, only limited colocalization could be appreciated with EEA1 as marker for the early endosomal compartment (Fig. 1 c). In contrast, vesicular podocin could be shown to partly reside in acidic organelles using fixed Lysotracker probes (Fig. 1d). Podocin demonstrated a more pronounced colocalization with co-expressed, Gfp-tagged CD63 (also known as LAMP3), a member of the tetraspanin superfamily localizing predominantly to the plasma membrane and vesicles of the late endosomal and lysosomal compartment (Fig. 1 e) [26]. We therefore concluded that a fraction of podocin resides in the late endosomal compartment positive for CD63/LAMP3. In agreement, the immunofluorescence signal of transiently expressed, V5-tagged podocin in podocytes revealed a similar pattern of intracellular and plasma membrane podocin distribution and co-localization with Gfp-tagged CD63/LAMP3 (Fig. 1f). Intracellular localization of podocin seems not to be influenced by N-terminal tagging as perfect colocalization of tagged and untagged podocin could be demonstrated and untagged podocin was also shown to localize to Gfp-CD63/LAMP3-positive vesicles (supplemental Fig. 1 a, b).

### The C-Terminus Regulates Plasma Membrane Fraction and Internalization of Podocin

**Figure 2:**
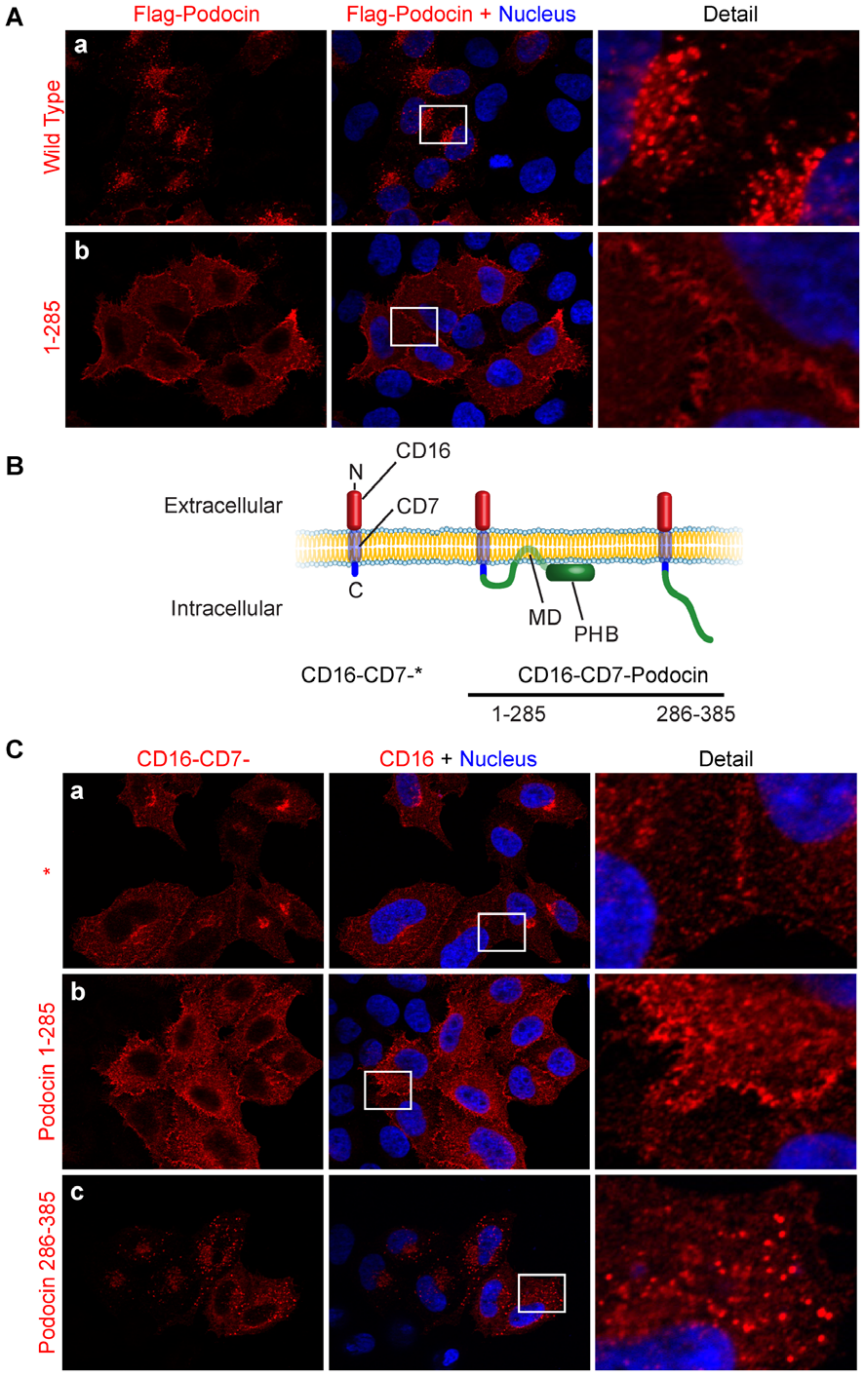
A C-terminal domain regulates plasma membrane localization of podocin. A. Immunofluorescence for a transiently expressed, Flag-tagged truncation of podocin (podocin^1–285^) reveals increased plasma membrane localization in comparison with podocin wild type in HeLa-cells (b and a respectively). B. Schematic representation of constructs consisting of the extracellular domain of CD16 and the transmembrane domain of CD7 fused to different parts of podocin. C. Immunofluorescence using anti-CD16 antibody showed primarily membranous staining patterns for CD-16-7-* and CD16-7-podocin^1–285^ (a, b). In contrast, immunofluorescence for CD16-7-podocin^286–385^ revealed multiple vesicular structures (c), thereby proving analogous localization of CD16-fusion constructs and V5/Flag-tagged constructs.

Various domains of podocin have been shown to mediate specific functions, such as interaction with nephrin, plasma membrane association and lipid raft binding [10], [27]. However, no function of the C-terminus distal from the PHB-domain has been described, although mutations in this region have been found in patients suffering from steroid-resistant nephrotic syndrome [28]. We hypothesized that this region, podocin^286–385^, might participate in the regulation of podocin localization. For a comparison of the subcellular localizations of podocin wild type and a truncation lacking the C-terminus, podocin^1–285^, we overexpressed these proteins in HeLa-cells. Strikingly, podocin^1–285^ localized in a distinct way from podocin wild type, staining mainly the plasma membrane and only few intracellular vesicles, suggesting a role of podocin^286–385^ in regulating podocin localization (Fig. 2A).

The analysis of podocin cell surface expression is complicated by the lack of extracellular domains. To address, whether increased plasma membrane expression was a result of reduced internalization rather than increased shuttling to the plasma membrane, we created constructs consisting of either podocin^1–285^ or podocin^286–385^ fused to the extracellular domain of human CD16 and the transmembrane domain of CD7 (schematic representation in Fig. 2B). We first compared the subcellular localization of these constructs in steady state to the V5/Flag-tagged constructs in order to evaluate their applicability for studying podocin trafficking. CD16-CD7-podocin^1–285^ localized in a fashion analogous to the Flag-tagged construct. CD16-CD7-podocin^286–385^ mainly localized to intracellular vesicles in contrast to our control construct CD16-CD7, which localized mainly to the plasma membrane, thereby supporting the hypothesis that podocin^286–385^ harbors an internalization promoting motif (Fig. 2C). A fusion construct of CD16-CD7 with podocin full length was mainly retained in the endoplasmatic reticulum and was not used for further studies (supplemental Fig. S2 a). For a dynamic analysis of podocin internalization, live HeLa cells expressing the aforementioned constructs were exposed to CD16 antibody in the cell culture media, thereby labeling only podocin-fusion constructs with their CD16-tag on the extracellular surface. After washing, cells were incubated at 37°C to allow internalization of the antibody or at 4°C for controls. After stripping remaining antibody from the extracellular surface, cells were fixed. No internalized fraction of CD16-CD7 alone or CD16-CD7-podocin^1–285^ could be detected after 20 minutes at either 4°C or 37°C (supplemental Fig. S2 b–e). In contrast, in cells expressing CD16-CD7-podocin^286–385^ vesicular structures were observed. No staining was detectable in 4°C controls (supplemental Fig. S2, f and g). These data indicate that podocin^286–385^ efficiently mediates a signal for internalization.

### Podocin^340–350^ Regulates Subcellular Localization of Podocin

**Figure 3:**
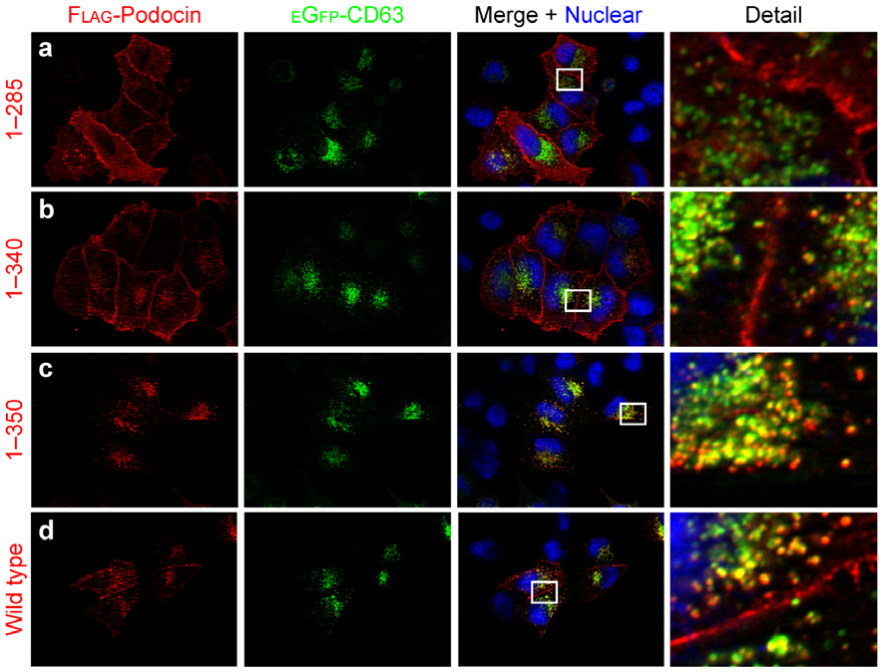
The domain podocin^340–350^ regulates the subcellular localization of podocin. A–D. Various truncations of Flag-tagged podocin were coexpressed with eGfp-tagged CD63 in HeLa-cells. Immunofluorescence using anti-Flag antibody revealed a primarily membranous staining pattern for podocin^1–285^ and podocin^1–340^ (a and b). In contrast, podocin^1–350^ and podocin wild type were shown to also localize to intracellular vesicles costaining with CD63 hinting at a crucial role of podocin^340–350^ in determining the subcellular localization of podocin (c and d).

In order to specify the region within podocin^286–385^ responsible for determining its subcellular localization, we stained various truncated versions of podocin in HeLa-cells. This approach revealed that the truncations podocin^1–310^, podocin^1–335^ and podocin^1–340^ all exhibited a pattern comparable to podocin^1–285^ (Fig. 3a, b; supplemental Fig. S3a, b), localizing primarily to the plasma membrane and bearing little colocalization with CD63. In contrast, the truncations podocin^1–350^, podocin^1–365^ and podocin^1–377^ exhibited a more vesicular pattern similar to podocin wild type, colocalizing significantly with CD63 (Fig. 3c,d; supplemental Fig. S3c,d). These results suggest a role of podocin^340–350^ in regulating podocin plasma membrane expression.

### Podocin^TVV339,340,341^ Regulates Localization and Degradation of Podocin through a Lipid Raft-Independent Mechanism

**Figure 4:**
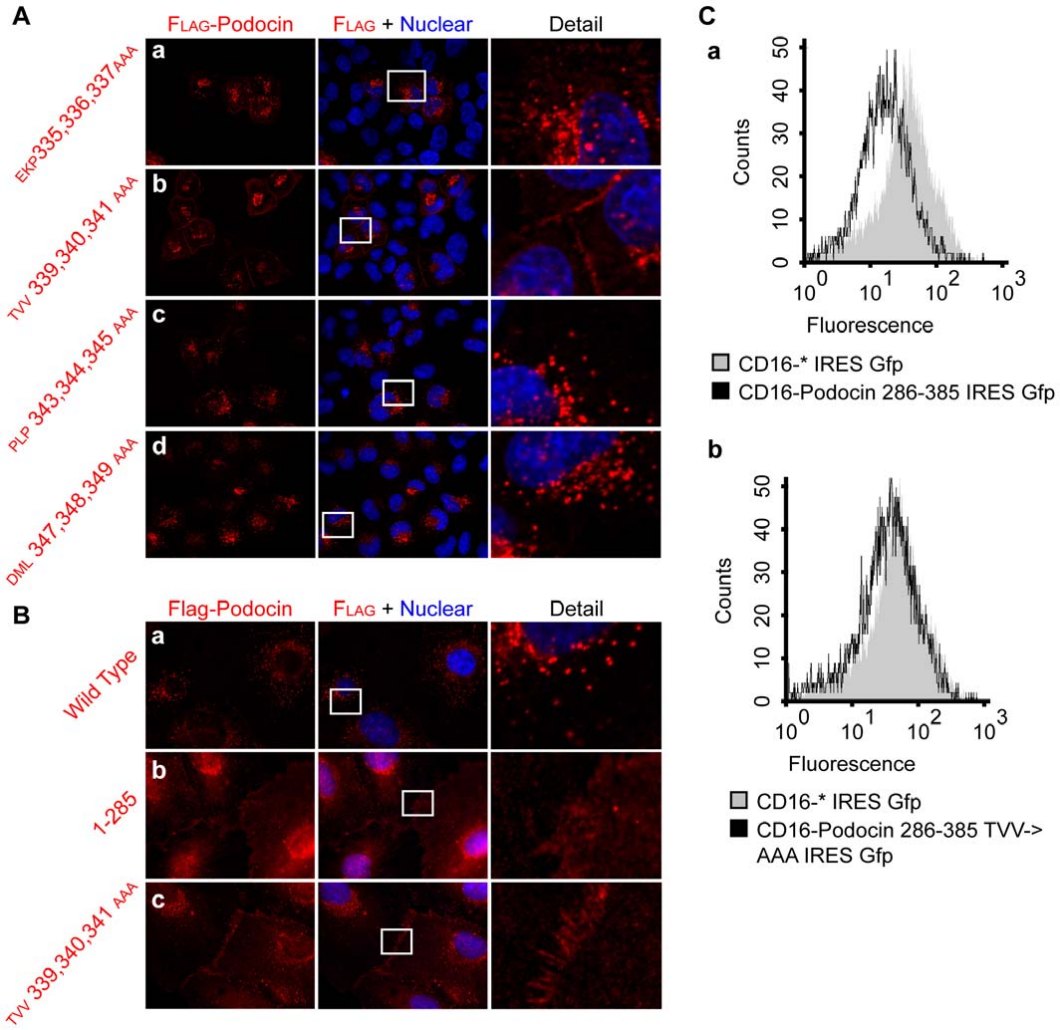
Podocin^TVV339, 340,341^ regulates the surface expression of podocin. A. Amino acid-triplets within podocin^335–350^ were mutated using a Quickchange approach. Mutated plasmids were transiently expressed in HeLa cells and localization of mutants was assessed by immunofluorescence using anti-Flag antibody. In contrast to the other mutants, podocin^TVV339,340,341AAA^ was shown to localize in a predominantly membranous pattern similar to podocin^1–285^ (a–d). B. Immunofluorescence of Flag-tagged podocin wild type, podocin^1–285^ and podocin^TVV339, 340,341AAA^ confirmed localization in transgenic differentiated podocytes to be analogous to HeLa cells (a–c). C. 293T-cells were transfected with plasmids expressing either CD16-7-*, CD16-7-podocin^286–385^ or CD16-7-podocin^286–385 TVV339,340,341AAA^ and cell surface expression was analyzed by FACS. As transfected cells also expressed Gfp driven from an internal ribosomal site from the same vector, gates were set to include Gfp-positive cells only. Cell surface expression of CD16-7-* and CD16-7-podocin^286–385TVV339,340,341AAA,^ was comparable (b), while less CD16-7-podocin^286–385^ could be detected at the plasma membrane, consistent with a role of podocin^TVV339,340,341^ in regulating internalization of podocin (a).

For a more precise mapping of the domain regulating podocin internalization we generated four Flag-tagged podocin variants with clusters of three amino acids mutated to alanine within podocin^335–350^. Immunofluorescence of these constructs overexpressed in HeLa cells revealed podocin^TVV339,340,341AAA^ to localize similarly to podocin^1–285^, whereas the other three mutants podocin^EKP335,336,337AAA^, podocin^PLP343,344,345AAA^ and podocin^DML347,348,349AAA^ exhibited a staining pattern comparable to podocin wild type (Fig. 4A). We confirmed the localizations of podocin wild type, podocin^1–285^ and podocin^TVV339,340,341AAA^ in transgenic podocytes stably expressing the construct to be analogous to the patterns seen in Hela cells (Fig. 4B). Thus, podocin^TVV339,340,341^ seems to be responsible for the regulation of plasma membrane expression of podocin. In order to quantify the difference in cell surface expression we used a FACS based approach. Cell surface expression of CD16-CD7-podocin^286–385^ ^TVV339,340,341AAA^ was comparable to a construct only consisting of CD16-CD7-*, while less cell surface signal could be detected with CD16-CD7-podocin^286–385^. This is consistent with a role of podocin^TVV339,340,341^ in regulating podocin plasma membrane localization (Fig. 4C).

**Figure 5:**
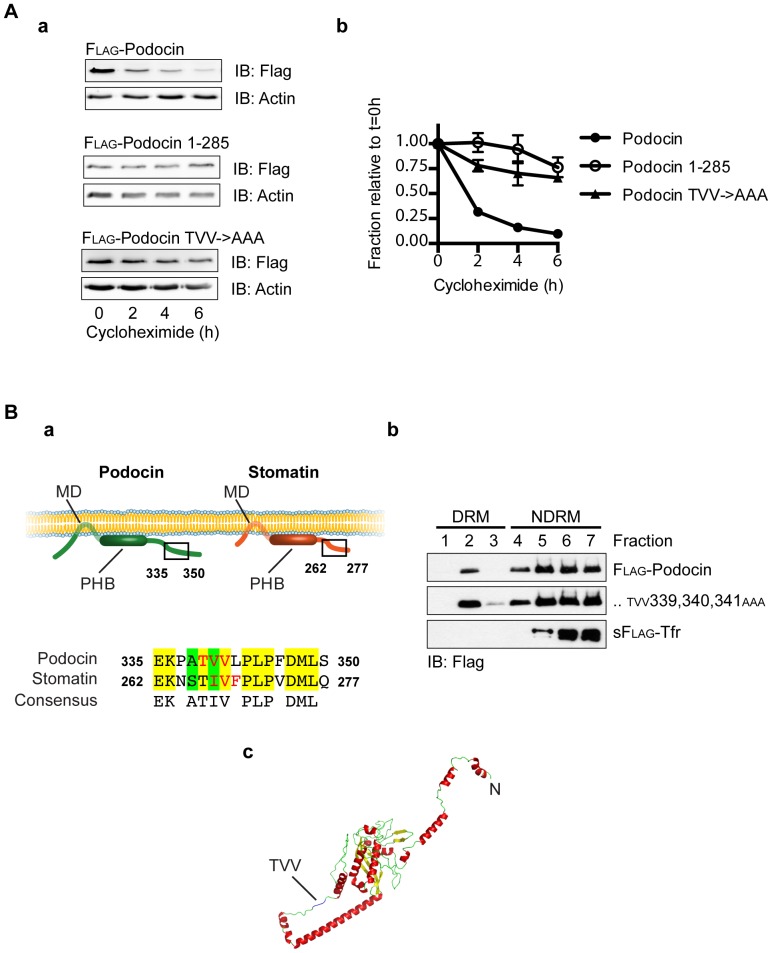
Podocin^TVV339,340,341^ regulates the turnover of podocin through a lipid raft-independent mechanism. A. Differentiated podocytes stably expressing Flag-tagged podocin, podocin^1–285^ or podocin^TVV339,340,341AAA^ were exposed to the translation inhibitor cycloheximide for the times as indicated and analyzed per western blot using anti-Flag antibody (a). Actin levels detected by anti-actin antibody served as loading control. Podocin^1–285^ and podocin^TVV339,340,341AAA^ were shown to be more stable than podocin wild type, consistent with a regulatory role of podocin^TVV339,340,341^ in its degradation. (b) Summarizes the results of three experiments. Podocin levels were normalized to actin levels. B. (a) shows a schematic comparison between the PHB-domain proteins podocin and stomatin. Umlauf et al. proved a motif partially overlapping with podocin^TVV339,340,341^ to play a crucial role in lipid raft binding. (b) HEK293T cells were transfected with the plasmids as indicated, lysed in 1% TX-100 on ice and subjected to flotation gradient centrifugation to prepare detergent-resistant membranes (DRM). In contrast to the control protein transferrin receptor, both podocin wild type and podocin^TVV339,340,341AAA^ were detected in DRM. C. Graphical representation of the structure prediction analysis of podocin using the I-Tasser algorithm revealed exposed position of podocin^TVV339, 340,341^.

Usually, plasma membrane associated proteins are internalized before degradation. Therefore, we also investigated into the half-life of podocin wild type versus podocin^1–285^ and podocin^TVV339,340,341AAA^. Experiments using the translation inhibitor cycloheximide allowed us to study degradation kinetics without interference due to podocin synthesis. Podocyte cell lines stably expressing either Flag-tagged podocin wild type, podocin^1–285^ or podocin^TVV339,340,341AAA^ were treated with cycloheximide for different time periods and analyzed for amount of podocin by western blotting. Indeed, podocin^1–285^ was much more stable over time, showing that its degradation is markedly reduced when compared with podocin wild type. Similarly, podocin^TVV339,340,341AAA^ showed a half life similar to podocin^1–285^, indicating that podocin^TVV339,340,341^ plays an essential role in the regulation of podocin degradation (Fig. 5A). These results were consistent with observations made using transiently transfected HEK293T cells (data not shown).

Interestingly, Umlauf and colleagues were able to show that the disruption of a motif in the podocin-related protein stomatin partially overlapping with podocin^TVV339,340,341^, stomatin^IVF267,268,269^ (schematic comparison in Fig. 5B, a), leads to the abrogation of lipid raft binding [29]. We therefore hypothesized that altered turnover kinetics after disruption of podocin^TVV339,340,341^ might be a result of defect lipid raft binding. However, in contrast to the control protein transferrin receptor, flotation gradient centrifugation revealed association of both podocin wild type and podocin^TVV339,340,341AAA^ with detergent resistant membranes, indicating intact recruitment of the mutant to lipid rafts (Fig. 5B, b).

In order to assess the localization of the TVV339,340,341 motif in the 3D configuration of podocin we performed a structure prediction analysis using the I-Tasser algorithm [24]. This showed podocin^TVV339,340,341^ to be exposed on the protein surface, thereby being accessible for presumptive interaction partners regulating podocin localization and degradation (Fig. 5B, c).

## Discussion

Spatiotemporal regulation of the slit diaphragm proteins nephrin and podocin is an essential mechanism in glomerular homeostasis and injury. Recent studies have focused on the mechanisms regulating the internalization of nephrin: PKCα was shown to mediate nephrin endocytosis via the regulation of an interaction between nephrin and β-arrestin2 [17], [30], [31]. In certain human glomerulopathies and in a CD2AP-deficiency model, nephrin internalization was also found to be stimulated by ubiquitination [16], [19], [32]. Moreover, Qin and co-workers proposed a raft-mediated endocytic pathway for nephrin following its phosphorylation [18]. However, no signals have been characterized mediating turnover cues for podocin.

We demonstrated that next to the plasmamembrane a considerable amount of podocin localizes to the late endosomal compartment, where it colocalizes with CD63/LAMP3 partially overlapping with acidic organelles. We then proposed a role of the C-terminus in regulating plasma membrane expression of podocin, suggested by markedly increased plasma membrane localization and reduced internalization of a truncation lacking the C-terminus. To further dissect the region mediating this effect we used several truncation variants as well as podocin Quickchange mutants in immunofluorescence based assays, a FACS-based approach and degradation assays to provide evidence that podocin^TVV339,340,341^ represents the domain responsible for mediating degradation signals and plasma membrane localization. Since podocin is a raft-associated molecule [10], a raft-dependent endocytic pathway has been suggested for nephrin [18] and a region of the podocin related protein stomatin partially overlapping with podocin^TVV339,340,341^ was revealed to mediate lipid raft binding [29], we tested for the association of podocin^TVV339,340,341AAA^ with lipid rafts. This revealed the presence of both podocin wild type and the mutant in lipid rafts, excluding the possibility of podocin^TVV339,340,341AAA^ exerting a change to turnover kinetics due to defect lipid raft binding. To support biological relevance consolidated findings obtained by use of easily manipulable cell lines like Hela and Hek293-T were validated in an immortalized podocyte cell line widely accepted as an appropriate cell culture system in podocyte cell biology. Being solely performed in *in vitro* cell culture systems, our data provide neither an *in vivo* proof of the biological relevance of the C-terminus and the TVV339,340,341 motif for podocin degradation and subcellular localization nor do they document the direct impact of podocin turnover on slit diaphragm physiology. Podocin trafficking could also be influenced by accessory podocin binding proteins *in vivo* that are absent from the cell culture systems used. However, these data provide several clues for further investigation into podocin function. First, it remains unclear whether the large fraction of podocin localizing to the late endosomal compartment is due to a high turnover rate of cell membrane-bound podocin or whether podocin might fulfill a specific purpose at the endosomal compartment, as proposed for other members of the PHB-domain protein family [33], [34]. Also, the exact route of internalization remains to be established. Being lipid raft-associated and bearing little colocalization with EEA1, a Clathrin-independent and raft-mediated, non-conventional endocytic pathway seems very likely, consistent with suggestions for endocytic trafficking of other PHB-domain proteins [34]. Intriguingly, the podocin-related protein flotillin-1 defines a clathrin-independent endocytic pathway [35] which could lead to the hypothesis that podocin not only assembles members of the slit diaphragm but also orchestrates its internalization via a self-defined pathway.

While no reports have been published on the regulation of podocin internalization, Tossidou and co-workers demonstrated that podocin was ubiquitinated in a CD2AP-deficiency model, and ubiquitination was increased through the interaction between podocin and the adaptor protein CIN85 [19]. However, the effect of ubiquitination on podocin endocytosis was not assessed. These data are consistent with our own unpublished observations that podocin is ubiquitinated. However, we did not observe altered subcellular distribution of a podocin variant with all lysine residues within podocin^285–385^ mutated to alanine (unpublished observations). Still, ubiquitination cannot be ruled out as a potential modifier of podocin turnover, especially taking into account that various mechanisms could be present that act depending on specific (patho-) physiological triggers. This could also hold true for nephrin, for which different mechanisms regulating its turnover have already been demonstrated [16], [17], [19].

Further research could also focus on the mechanism through which podocin^TVV339,340,341^ mediates the signal for degradation. While phosphorylation of the threonine within the motif could be one option, the divaline based motif could possibly have a comparable effect to a dileucine based motif, which is known to mediate internalization signals [36]. Moreover, a systematic screening approach comparing podocin wild type and the mutant could yield novel binding partners constituting the turnover machinery.

In summary, this study identified a previously uncharacterized domain regulating the localization and degradation of podocin. Disruption of a three amino acids-comprising motif led to increased plasma membrane expression and reduced degradation of podocin through a lipid raft-independent mechanism. Relating this mechanism to turnover events in glomerular maintenance and injury could lead to an extended understanding of proteinuric diseases and provide novel therapeutic options.

## Supporting Information

Figure S1 **N-terminal tagging of podocin does not seem to influence its subcellular localization in HeLa cells as there is a perfect overlap with overexpressed untagged podocin stained with a podocin specific antibody.** Untagged podocin also displayed significant colocalization with eGFP tagged CD63/LAMP3 (a and b respectively).
(TIF)

Figure S2 **A C-terminal domain regulates the internalization of podocin.** HeLa cells transiently expressing the constructs as indicated were incubated on ice with anti-CD16 antibody. Following a 20 minute incubation period with medium at 4°C versus 37°C remaining extracellular antibody was stripped and the cells were fixed and permeabilized. Immunofluorescence revealed no internalized fraction for control cells at 4°C (b, d, f). In contrast to both CD16-7-* and CD16-7-podocin^1–285^ (c and e) an internalized fraction could be detected with podocin^286–385^ after incubation at 37°C (g). A construct of CD16-CD7 fused to podocin wild type full length stained with anti-CD16 in permeabilized cells revealed a staining pattern indicating retention in the endoplasmatic reticulum (a).
(TIF)

Figure S3 **Subcellular localization of different podocin truncations.** A–D. Various truncations of Flag-tagged podocin were coexpressed with eGfp-tagged CD63 in HeLa-cells. Immunofluorescence using anti-Flag antibody revealed a primarily membranous staining pattern for podocin^1–310^ and podocin^1–335^ similar to podocin^1–285^ (a and b). In contrast, podocin^1–365^ and podocin^1–377^ were shown to localize similarly to podocin wild type (c and d).
(TIF)
