# Supplementary figures and images for: A Novel Domain Regulating Degradation of the Glomerular Slit Diaphragm Protein Podocin in Cell Culture Systems
Source: PLoS One. 2013 Feb 20;8(2):e57078. doi: 10.1371/journal.pone.0057078 (PMC3577791; doi:10.1371/journal.pone.0057078)

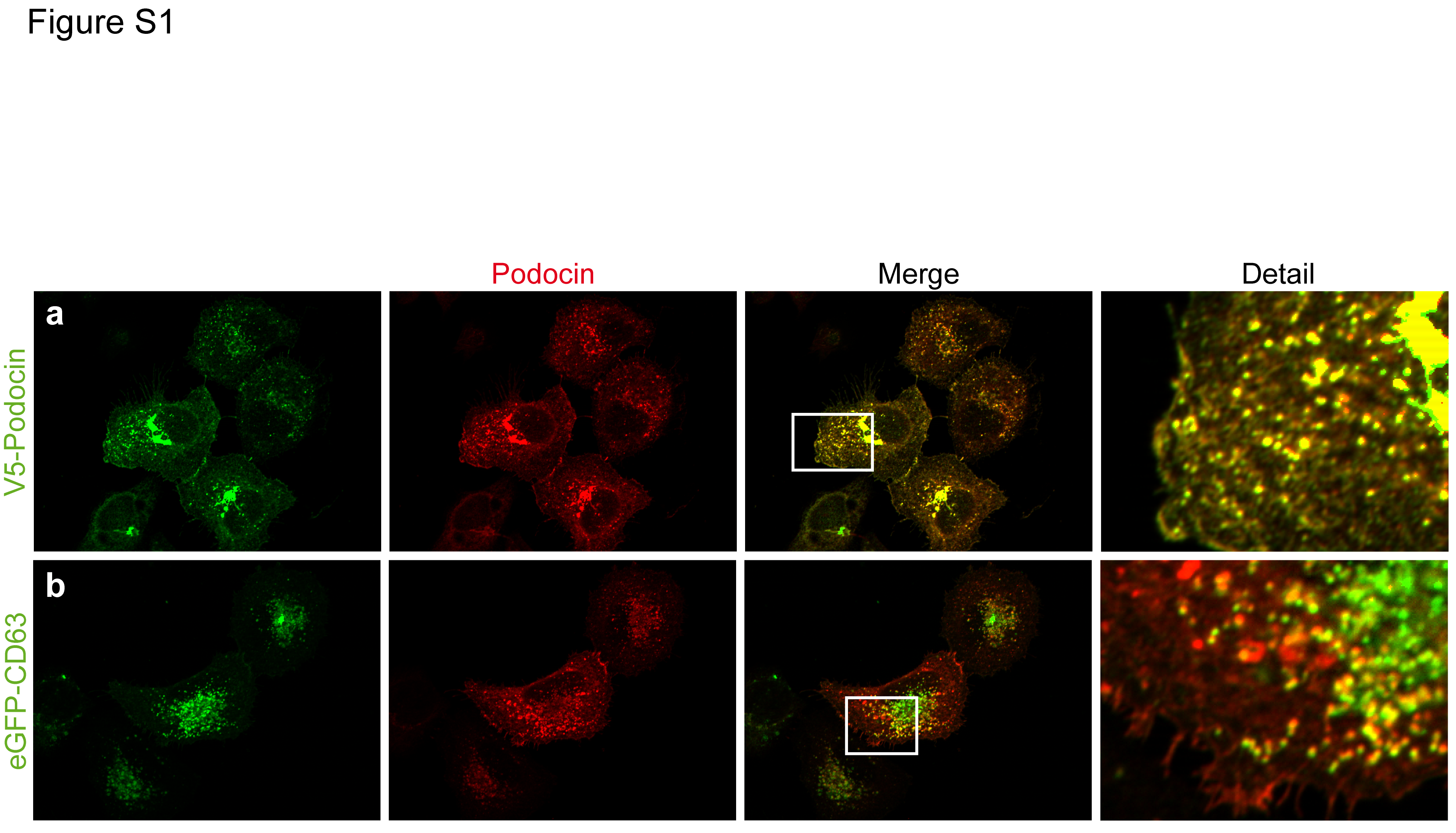

Supplement: Figure S1 — N-terminal tagging of podocin does not seem to influence its subcellular localization in HeLa cells as there is a perfect overlap with overexpressed untagged podocin stained with a podocin specific antibody. Untagged podocin also displayed significant colocalization with eGFP tagged CD63/LAMP3 (a and b respectively). (TIF) [file pone.0057078.s001.tif]

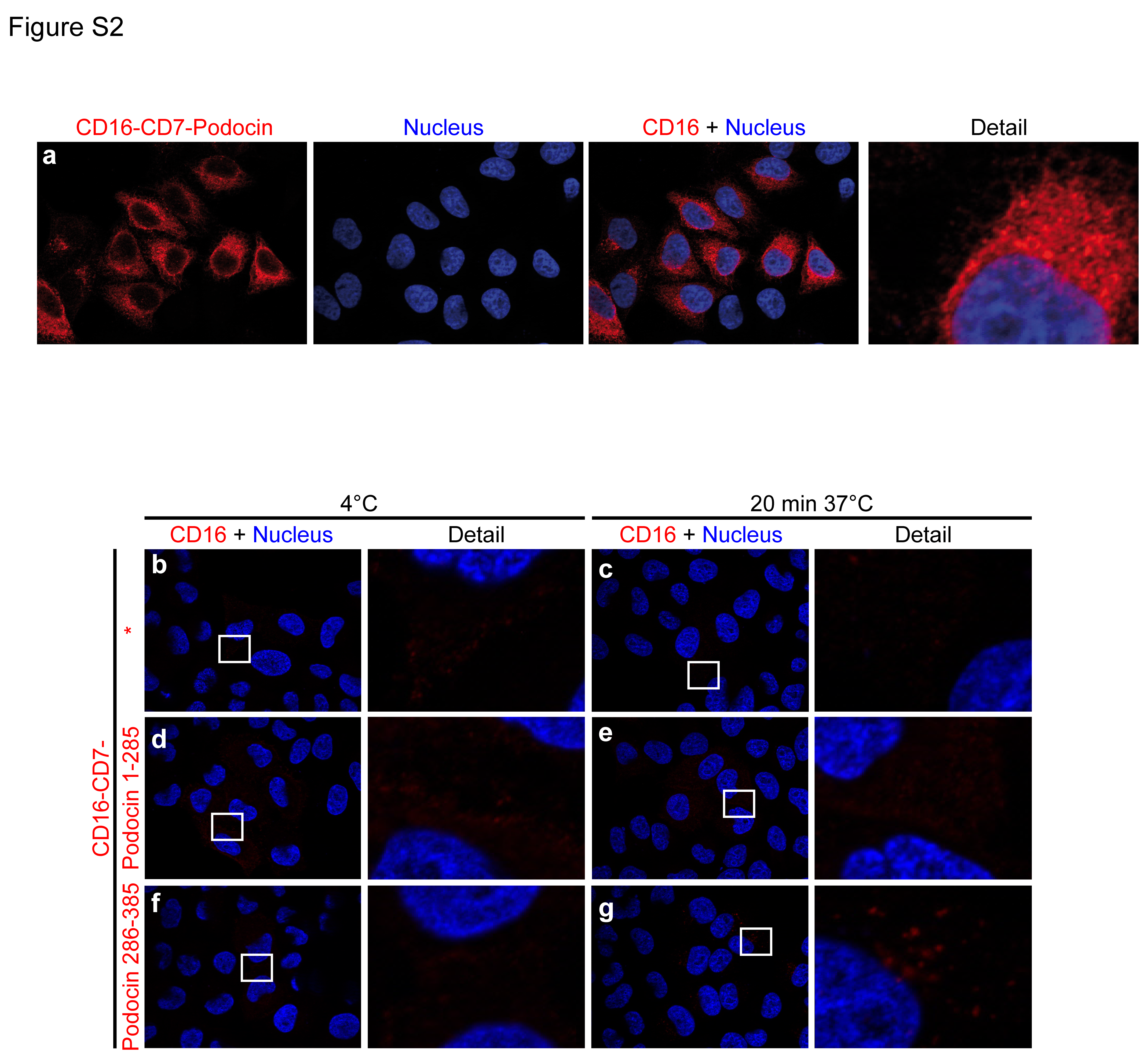

Supplement: Figure S2 — A C-terminal domain regulates the internalization of podocin. HeLa cells transiently expressing the constructs as indicated were incubated on ice with anti-CD16 antibody. Following a 20 minute incubation period with medium at 4°C versus 37°C remaining extracellular antibody was stripped and the cells were fixed and permeabilized. Immunofluorescence revealed no internalized fraction for control cells at 4°C (b, d, f). In contrast to both CD16-7-* and CD16-7-podocin1–285 (c and e) an internalized fraction could be detected with podocin286–385 after incubation at 37°C (g). A construct of CD16-CD7 fused to podocin wild type full length stained with anti-CD16 in permeabilized cells revealed a staining pattern indicating retention in the endoplasmatic reticulum (a). (TIF) [file pone.0057078.s002.tif]

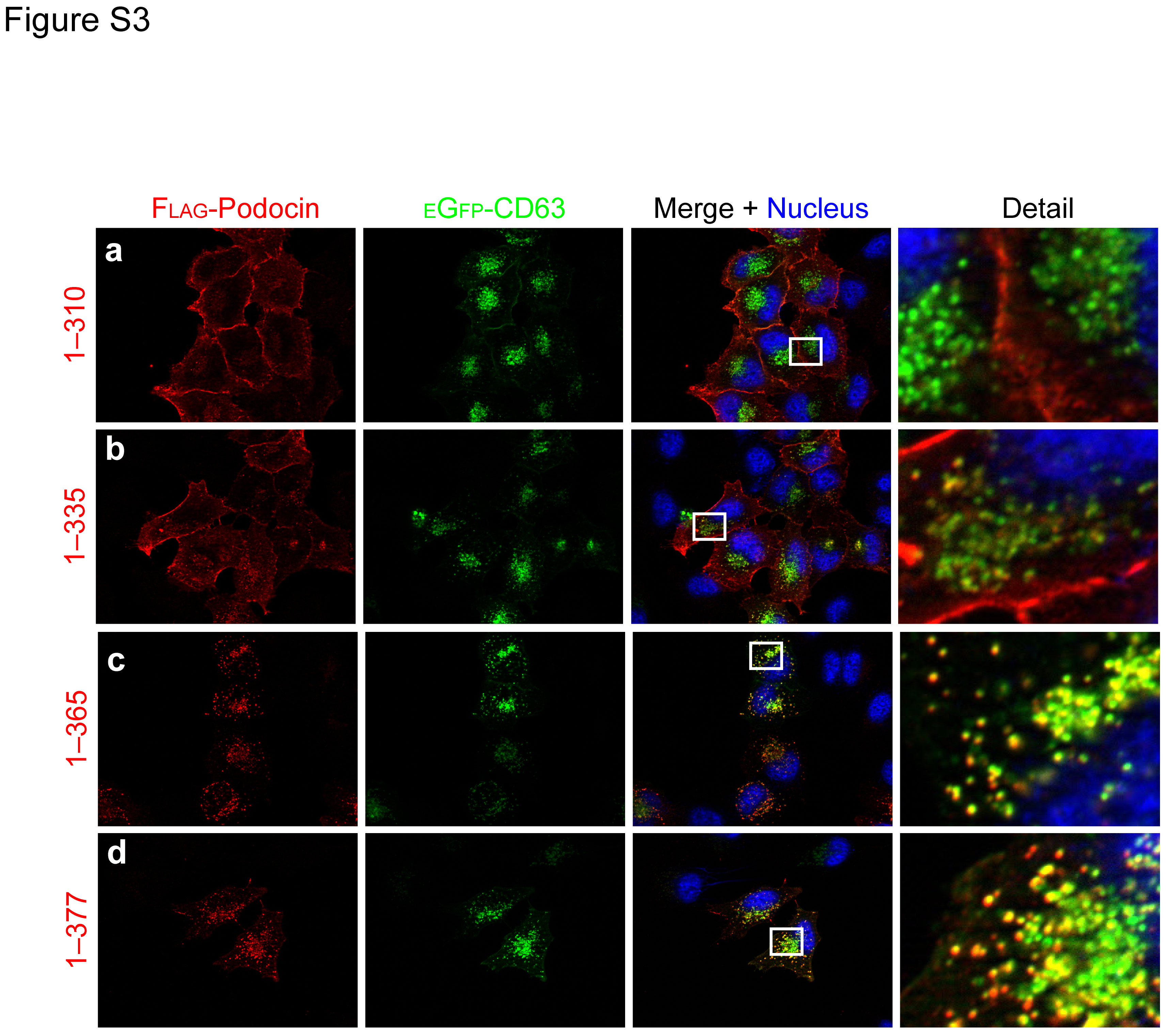

Supplement: Figure S3 — Subcellular localization of different podocin truncations. A–D. Various truncations of Flag-tagged podocin were coexpressed with eGfp-tagged CD63 in HeLa-cells. Immunofluorescence using anti-Flag antibody revealed a primarily membranous staining pattern for podocin1–310 and podocin1–335 similar to podocin1–285 (a and b). In contrast, podocin1–365 and podocin1–377 were shown to localize similarly to podocin wild type (c and d). (TIF) [file pone.0057078.s003.tif]
